# ﻿Geographic differentiation in male calling song of *Isophyamodestior* (Orthoptera, Tettigoniidae, Phaneropterinae)

**DOI:** 10.3897/zookeys.1122.85721

**Published:** 2022-09-26

**Authors:** Slobodan Ivković, Dragan Chobanov, Laslo Horvat, Ionuț Ștefan Iorgu, Axel Hochkirch

**Affiliations:** 1 Trier University, Department of Biogeography, Universitätsring 15, 54296 Trier, Germany Trier University Trier Germany; 2 Institute of Biodiversity and Ecosystem Research, Bulgarian Academy of Sciences, Tsar Osvoboditel Blvd. 1, 1000 Sofia, Bulgaria Institute of Biodiversity and Ecosystem Research, Bulgarian Academy of Sciences Sofia Bulgaria; 3 Ruwerer Str. 39, 54292 Trier, Germany Unaffiliated Trier Germany; 4 “Grigore Antipa” National Museum of Natural History, Kiseleff Blvd. 1, 011341 Bucharest, Romania “Grigore Antipa” National Museum of Natural History Bucharest Romania

**Keywords:** Balkans, bioacoustics, bush-cricket, oscillogram, Pannonian Basin, stridulatory file

## Abstract

We studied the songs and morphology of the stridulatory file of *Isophyamodestior* across its complete geographic range, in order to test our hypothesis that the male calling song of the species shows strong differentiation between the northern (Pannonian) and southern (Balkan) parts of its distribution range, reflecting its disjunct distribution. Our analyses confirm this hypothesis, separating analyzed specimens of *I.modestior* into two main groups - one present in the central part of the Balkan Peninsula (representing *Isophyamodestiorsensu stricto*), with the second group occurring in the Pannonian Basin, Dinarides, Slovenia and NE Italy. The most reliable difference between the groups is the duration of the main syllable, the number of stridulatory teeth and number of pulses in the main syllable, where all values are higher in specimens from the Balkan Peninsula. Additional analyses showed that within the second group, there are differences in analyzed characters between specimens from the Pannonian Basin and specimens from the Dinaric area, the latter ones having intermediate song characteristics, closer to the group from the Balkan Peninsula. Our study shows that detailed bioacoustic analyses can help to unravel patterns of intraspecific differentiation and thus provide a useful tool for taxonomic studies.

## ﻿Introduction

Acoustic signals are a major channel for many animal species in order to provide various information (Bradbury and Vehrencamp 2011). Such signals can contain detailed information about the individual’s identity, the position of the singing animal, body size, age, or physiological condition. Acoustic communication has been studied in many animal groups, such as fishes (Tricas and Boyle 2014), frogs (Xie et al. 2018; Filer et al. 2021), birds (Ng and Rheindt 2016), mammals (Bernstein et al. 2016) and many arthropods (Gogala and Popov 1997; Sweger and Uetz 2016). In crickets and katydids, acoustic communication is widespread and has, therefore, been studied for many decades (Faber 1928; Jacobs 1953). Fossils of Orthoptera possessing stridulatory organs exist from the Triassic and Jurassic periods (Béthoux and Nel 2002; Gorochov and Rasnitsyn 2002; Senter 2008), placing them among the first organisms with fossilized sound-producing structures.

Bioacoustic communication is one of the main characteristics of mating systems among Orthoptera (Hall and Robinson 2021). In general, male orthopterans produce a calling song and, in some groups, females respond acoustically to the male (Heller and von Helversen 1986; Heller et al. 2018). In the majority of the male Tettigoniidae (bush-crickets), the acoustic signal is produced with the stridulatory organ, which is placed at the base of the tegmina, where a toughened edge (*plectrum*) of the right tegmina is scraped against a file of teeth (*pars stridens*) on the underside of the left tegmina (Ragge and Reynolds 1998). The calling songs and the morphology of the stridulatory files are known to be reliable traits for the identification of a cryptic species that may otherwise be very difficult to identify due to high morphological similarity (Heller et al. 2004; Orci et al. 2005).

Zhantiev and Dubrovin (1977) published the first study on the stridulatory morphologies and song patterns in the genus *Isophya* Brunner von Wattenwyl, 1878, establishing that both characters are strictly species-specific within the genus and effective in discriminating cryptic species. These results were confirmed by Heller (1988) and Heller et al. (2004), resulting in the description of various new cryptic taxa within the genus *Isophya* (Orci et al. 2010; Iorgu 2012; Iorgu et al. 2017; Sevgili 2020) and resolving some identification problems in widely distributed species (Heller 1988).

**Figure 1.**
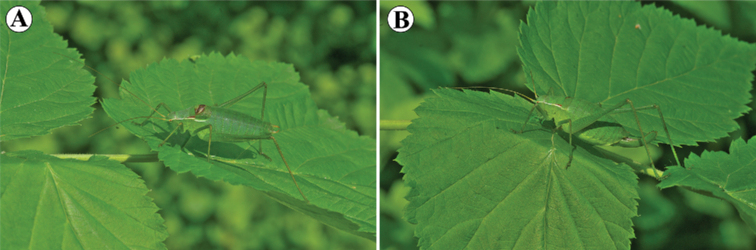
Habitus of *Isophyamodestior***A** male (Serbia: Beočin, 19 V 2018) **B** female (Serbia: Beočin, 19 V 2018).

**Figure 2.**
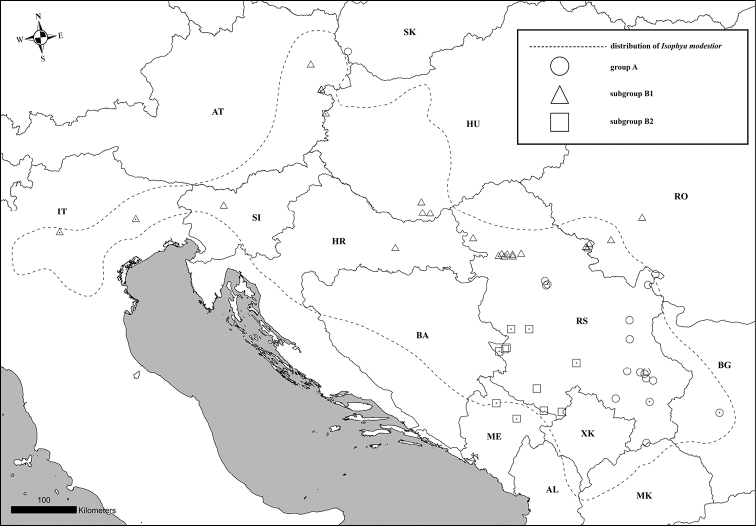
Distribution map of *Isophyamodestior* (dotted line from Hochkirch et al. 2016) showing the localities from where songs were analyzed (for detailed locality data see Suppl. material 1); white symbol with black dot literature data; white symbol new, unpublished data. Groups are defined on the basis of the differentiation in the analysed characters (male calling song and morphology of the stridulatory file).

*Isophyamodestior* Brunner von Wattenwyl, 1882 is an interesting model species to study intraspecific variation in song and morphology of the stridulatory file across its range, as it has a disjunct distribution and shows some substantial morphological variability (Heller 1988; Heller 1990; Ingrisch 1991; Heller et al. 2004; Ingrisch and Pavićević 2010; Ingrisch and Pavićević 2012; Chobanov et al. 2013; Brizio et al. 2020). The body color of the species is green with white side keels on the pronotum and the reddish band on the upper part (Fig. 1). The male fore wing is green with dark spots and they are usually as long as the pronotum. Cerci are strongly curved in their distal third, while ovipositor is slightly curved. *I.modestior* usually inhabits bushes on the forest edges and clearings, but also can be found on mesic and semi-dry grasslands. The species is widespread in Europe, occurring from eastern Austria and northern Italy through to Bulgaria, but the distribution range is separated by a gap into two parts. The southern part of its distribution is in the NW Balkans (SW Romania, NW Bulgaria, N North Macedonia, inland part of Montenegro, Bosnia and Herzegovina, Kosovo, N Albania and Serbia), while the northern part of the range covers scattered populations in Croatia, Hungary, Slovakia, Austria, Slovenia and NE Italy (Chobanov et al. 2013; Hochkirch et al. 2016; Krištín et al. 2020) (Fig. 2).

We hypothesized that the male calling song of *I.modestior* shows strong differentiation between the northern (Pannonian) and southern (Balkan) parts of its range, reflecting its disjunct distribution. Therefore, we studied songs and the morphology of the stridulatory file of *I.modestior* across its complete range, complemented by data available in the literature. We analyzed the bioacoustic and morphological data for intraspecific differentiation.

## ﻿Material and methods

### ﻿Sample collection

Adults of *Isophyamodestior* were collected in natural habitats throughout their range (Fig. 2) and brought to the laboratory, where their songs were recorded. In some instances, specimens were collected as nymphs, matured in a laboratory and recorded several days after maturation. Besides our own data, we used all published data on the song of the species (Heller 1988; Ingrisch 1991; Fontana 1998; Fontana et al. 2002; Nagy et al. 2003; Heller et al. 2004; Orci et al. 2005; Ingrisch and Pavićević 2010; Ingrisch and Pavićević 2012; Iorgu and Iorgu 2012; Chobanov et al. 2013; Brizio et al. 2020) and recordings deposited at Orthoptera Species File (Cigliano et al. 2021).

For our own sound recordings, we used Roland R-05, Edirol R-09HR and ZOOM H2 digital audio recorders. The majority of recordings were made at night, when the males actively sing. Sound analyses and oscillograms were made with Adobe Audition CC 2015 and Audacity. Parts of the stridulatory files were studied with a scanning electron microscope (JEOL JSM 6460 LV) at the UCEM-NS (University Center for Electron Microscopy, Novi Sad), while other material was studied under a stereomicroscope.

### ﻿Bioacoustic terminology

In this study, we follow the terminology by Heller et al. (2004) and Ragge and Reynolds (1998):

- Calling song: spontaneous song produced by an isolated male.
- Main syllable duration: the sound produced by one complete up (opening) and down (closing) stroke of the tegmina.
- Pulse: a simple, undivided, transient train of sound waves (here: the highly damped sound impulse arising as the impact of one tooth of the stridulatory file).
- After-click: pulse produced with considerable delay after the main pulse group.

Since most of our recordings were more than one hour long, we analyzed 5–10 minutes of each recording after which characteristics in 10 syllables per specimen were chosen as an unbiased random sample. Detailed data on song recordings presented in this paper are provided in the Suppl. material 1.

Two published songs were excluded from this study:

1. Gomboc and Šegula (2014): the oscillogram does not match *I.modestior*;
2. Heller et al. (2004): inaccurate locality (either 20 km southwest of Ptuj or Kocara (Kozara?) national park (northern Bosnia).

In total, calling songs of 64 specimens from the complete distribution range were analyzed (Suppl. material 1, Fig. 2). For our study, we focused on the following characters: main syllable duration, number of pulses in main syllable and number of stridulatory teeth. To visualize the relationship between analyzed characters and populations, we ran a PCA (Principal Component Analysis) using the missMDA package in R (Version 4.1.2; R Core Team 2021). Based upon the results of preliminary studies, we assigned each individual to two groups (see results) and analyzed the three main characters for significant differences among those groups using ANCOVAs with temperature as covariate. To adjust the data to the model assumptions, we transformed the data using Box-Cox transformation using the MASS package (Venables and Ripley 2002). To test for song similarity in a multivariate context, we used Fisher’s discriminant analysis (FDA) calculated in the mda package for R (Hastie and Tibshirani 2020). Due to a high linear correlation between the number of stridulatory teeth and pulses (R^2^ = 0.72), we ignored the number of stridulatory teeth and deleted all individuals with missing data in one of the song traits.

## ﻿Results

**Figure 3.**
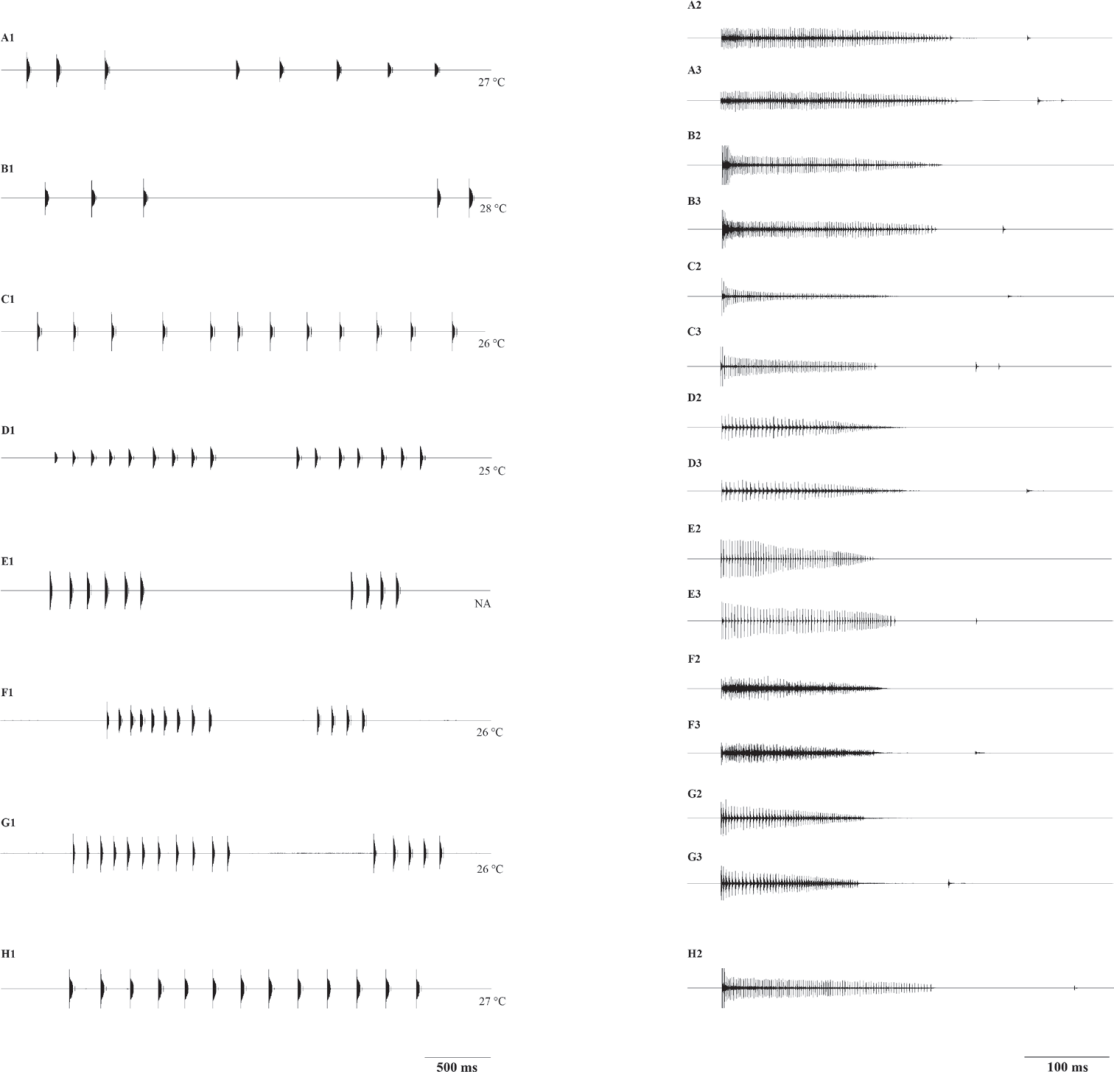
Oscillograms of the male calling song in *Isophyamodestior* across the range. Two groups (Group A and Group B) and two more subgroups (Subgroup B1 and Subgroup B2) can be separated on the basis of the calling song. Group A includes oscillograms **A** Lalinac (eastern Serbia, close to type locality) **B** Pinosava (central Serbia); Subgroup B1 includes oscillograms **C** Beočin (Pannonian Serbia) **D** Gudurica (Pannonian Serbia) **E** Carasova (Romania) **F** Mecsek (Hungary) **G** Loipersbach (Austria); Subgroup B2 includes oscillograms **H** Đerekare (Dinaric Alps, Serbia).

The calling song was relatively similar among populations and consisted of a train of single diminuending syllables, usually repeated in short sequences composed of two, rarely more than 13 syllables (Fig. 3). After-clicks were present in some specimens, varying from one to two, rarely three after-clicks (Fig. 3: A2, A3, B3, C2, C3, D3, E3, F3, G3 and H2).

**Figure 4.**
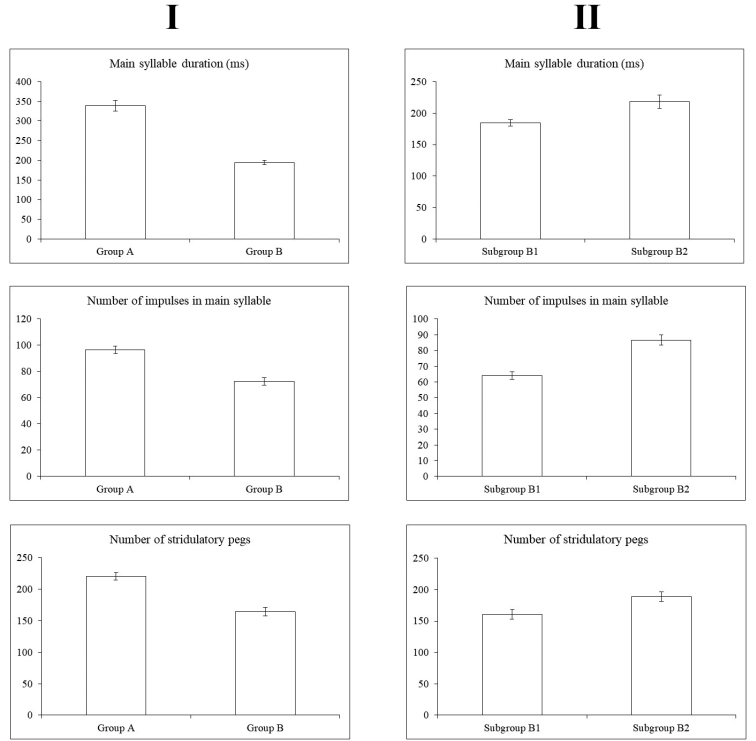
Variability in analyzed characters between the **A** and **B** groups (I) and B1 and B2 subgroups (II).

The two groups (Balkan vs. Pannonian) differed mainly in:

1. the duration of the main syllable;
2. the number of teeth on a stridulatory file in left tegmina;
3. the number of pulses in the main syllable (Fig. 4I).

Individuals belonging to Group A were found in Bulgaria, eastern and central Serbia. Individuals belonging to Group B were found in the Pannonian Basin (Pannonian Serbia, Romania, Hungary, Croatia, Austria and Slovakia,), Italy, Montenegro and western Serbia (Dinarides) (Fig. 2). The song of the specimen from Slovenia, which showed characters of both groups, was omitted from the statistical analyses presented in Fig. 4, but was included in PCA analyses (Fig. 7) in order to illustrate its position in a multidimensional context.

**Figure 5.**
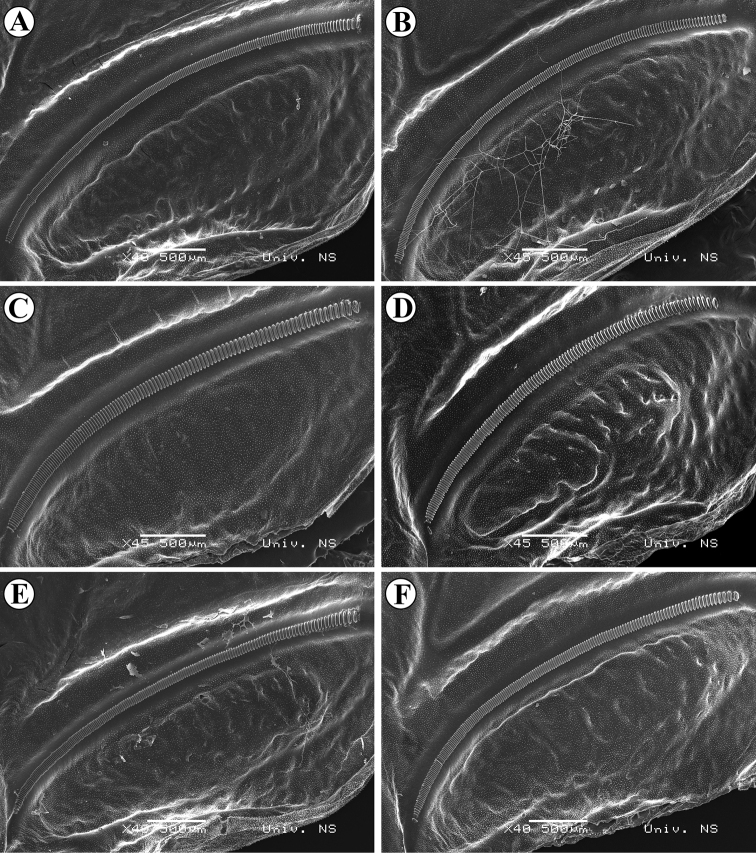
SEM photos of stridulatory files of different groups: Group A **A** Bancarevo (eastern Serbia, type locality) **B** Kladovo (north-eastern Serbia); Subgroup B1 **C** Mesić (Pannonian Serbia) **D** Deronje (Pannonian Serbia); Subgroup B2 **E** Durmitor, Pirlitor (Montenegro / not included in analyzes) **F** Ovčar-Kablar Gorge (western Serbia / not included in analyzes).

**Figure 6.**
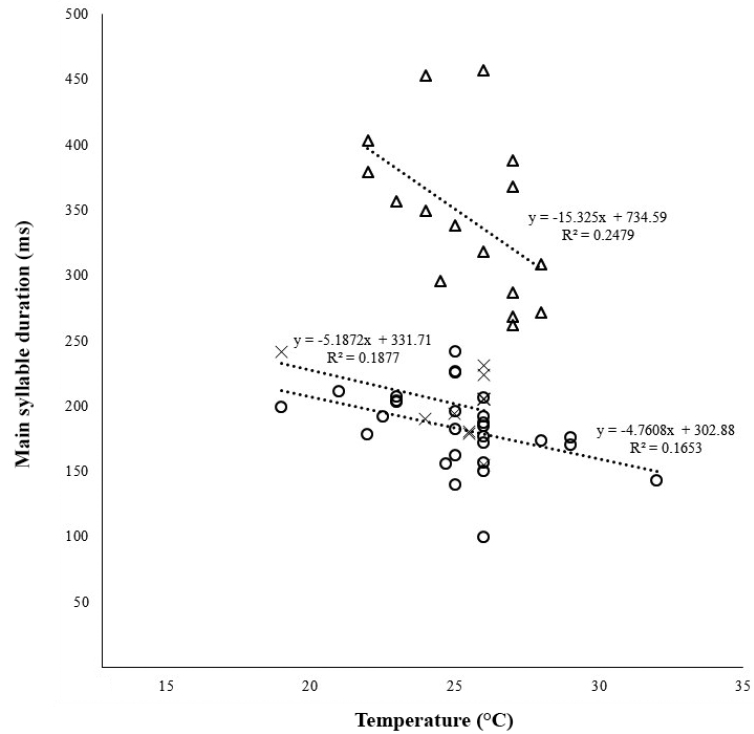
Two-dimensional scatter plot showing the temperature dependence of the main syllable duration between two groups (triangle Group A; circle Group B1; X Group B2).

In Group A, the main syllable consisted on average of 96.57 ± 2.86 pulses lasting for 338.6 ± 14.06 ms, which corresponds also with another character, the higher number (221 ± 5.61) of stridulatory teeth on a stridulatory file (Figs 5A, 5B) compared to Group B with 164.38 ± 7.02 teeth (Fig. 5C–F). The main syllable in Group B consisted of 72.38 ± 2.80 pulses and lasted 195.01 ± 5.35 ms. The groups differed significantly in syllable length (ANCOVA, λ = -0.4, F_1,49_ = 117.3, P < 0.001). There was a significant negative correlation between temperature and syllable length (ANCOVA, λ = -0.4, F_1,54_ = 10.66, P = 0.002). Both groups responded to temperature in a similar manner (ANCOVA, λ = -0.4, F_1,54_ = 0.03, P = 0.85), but were clearly separated by their syllable duration (Fig. 6).

Additional analyses within Group B showed that specimens could be subdivided into two subgroups (Fig. 4II): B1–Pannonian Basin (northern Serbia, Romania, Hungary, Croatia and Austria) and NE Italy; B2–Montenegro and western Serbia (Dinarides). The main syllable in Subgroup B1 consisted of 64.11 ± 2.52 pulses lasting for 184.42 ± 5.18 ms, while in Subgroup B2 the main syllable consisted of 86.67 ± 3.19 pulses lasting for 218.46 ± 10.60 ms. Furthermore, the number of stridulatory teeth (Figs 5C, 5D) was lower in Subgroup B1 (160.55 ± 7.71) than in Subgroup B2 (188.67 ± 7.86) (Figs 5E, 5F).

**Figure 7.**
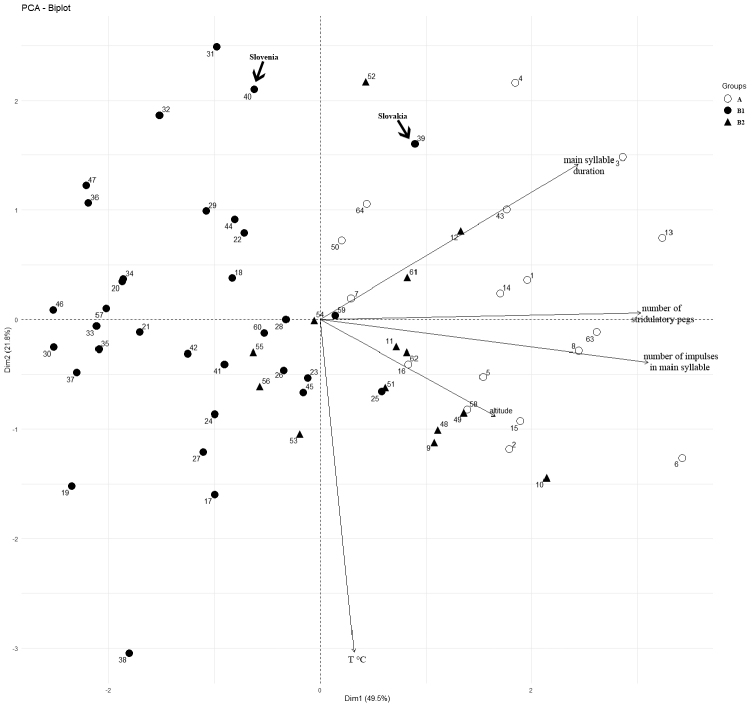
The Principal component analysis (PCA) showing the relationship between analyzed characters and populations (arrow indicates specimens from Slovenia and Slovakia).

The principal component analysis (PCA) illustrates a positive correlation of the number of stridulatory teeth, and number of pulses in the main syllable and syllable duration, all of which have strong loadings on the first axis (Fig. 7). While high numbers in all these characters specify group A, group B shows lower values. These analyses show that specimens with main syllable durations ≤ 250 ms, number of pulses ≤ 87 and number of stridulatory teeth ≤ 200 belong to group B, while specimens in group A have higher values. Dinaric specimens (subgroup B2) were placed at the upper edge of characters in group B, close to the group A, with the main syllable duration within the range 179–314.7 ms, number of pulses 66.2–94.5 and number of stridulatory teeth 178–204.

**Figure 8.**
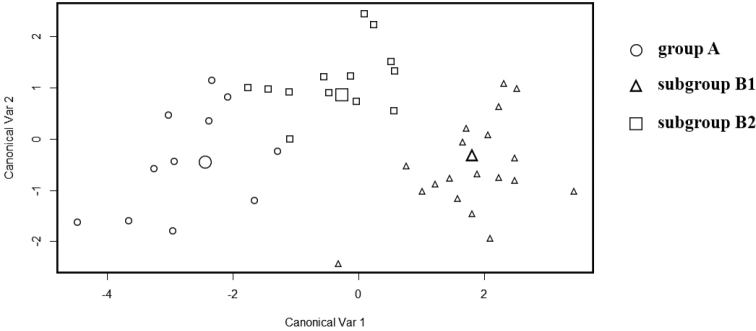
Plot of the two first canonical variables of a fda analysis on the song parameters. Large symbols represent groups centres.

The FDA showed that it is possible to discriminate between all three groups in a multivariate context (Fig. 8), with 83.7% of the predicted class memberships being correct. Within group B, 96.8% of all specimens were assigned to the correct class, while within group A, 83.3% were correctly assigned. Assignments to the subgroups of group B were less accurate, with 88.9% correctly assigned to group B1 and 76.9% to group B2.

## ﻿Discussion

Our analyses confirm the substantial geographic variation of song characteristics in *Isophyamodestior*, separating all samples into two main groups. The first group (A) is distributed on the Balkan Peninsula and represents *Isophyamodestiorsensu stricto*, while the second group (B) occurs in the Pannonian Basin, Dinarides and NE Italy. The most reliable difference between the groups is the duration of the main syllable, with group A showing a longer duration, but also a higher number of stridulatory teeth and higher number of pulses in the main syllable than group B. The song of the specimen from Slovenia showed characters of both groups, but the principal component analysis resolved its position in group B, which was presumed based on its locality. Altogether, the two groups showed a clear geographic pattern. The only exception is the specimen from Slovakia (39), which is closer to group A in the PCA plot, while geographically it should belong to group B. This individual had indeed a long main syllable (356 ± 17.05 ms), which is characteristic for group A. However, regarding the number of pulses in the main syllable (86.7 ± 2.58), the specimen fits better in group B. As this specimen was recorded very late in the season (28 July 2016), this might explain the longer duration of the main syllable, since temperature and age are known to have a strong impact on song duration in *Isophya* (Chobanov et al. 2013).

**Figure 9.**
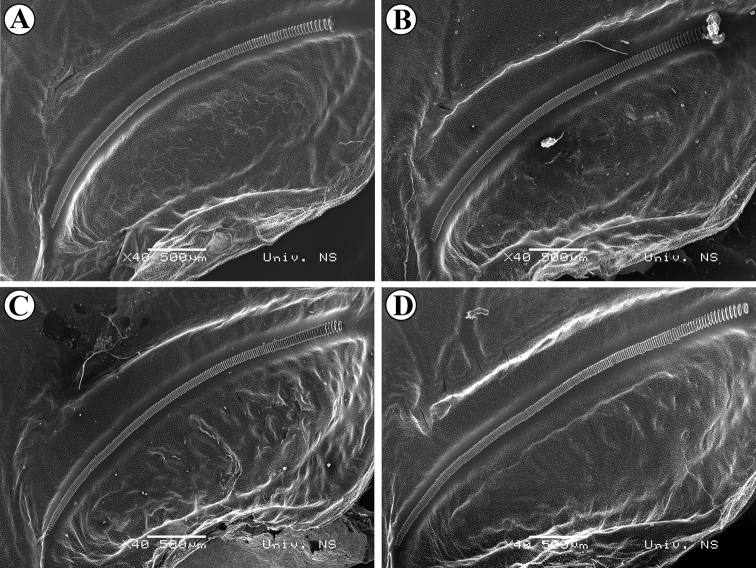
SEM photos of stridulatory files of different specimens from same population (Pannonian Serbia, Fruška Gora Velika Remeta) **A** 192 stridulatory teeth **B** 197 stridulatory teeth **C** 210 stridulatory teeth **D** 239 stridulatory teeth.

Within group B, the position of two specimens (25 and 59 – Fig. 7) is probably a result of their high number of stridulatory teeth (Fig. 9, see also Suppl. material 1), which was not observed in other analyzed populations. Furthermore, within group B, differences are also present between specimens from the Pannonian Basin (B1) and specimens from the Dinaric area (B2), the latter ones having intermediate song characteristics, closer to group A.

Due to the high variation of song characteristics within group B, our analyses do not allow to conclude whether the geographic structuring of bioacoustic parameters of *Isophyamodestior* is a matter of intraspecific variation or whether it reflects the existence of a cryptic species complex. On the other hand, our results support a stronger differentiation between group A and B. Those are mostly split geographically by the Sava-Lower Danube line. Rivers can represent significant barriers for flightless terrestrial insects with low mobility, but also climate-driven vicariances at a local scale could promote lineage diversification in *Isophya* (e.g., Chobanov et al. 2017). For example, the dry conditions in the lowlands of the Balkan Peninsula during Pleistocene Glacial maxima (Wright et al. 2003) may have promoted short-term speciation in mesophilic bush-crickets (Borissov et al. 2021).

Even though bioacoustic analyses represent a strong tool for identification of most of the species within genus *Isophya* (Heller et al. 2004), song parameters within this group may also be a matter of homoplastic/convergent evolution (Chobanov et al. 2017). Orthoptera songs are under strong selection, not only from inter- and intrasexual competition (female choice; e.g. Heller and Hemp 2020), but also from natural selection, e.g. by attracting predators or parasites (e.g. Wagner 1996). Therefore, the result of the present study may be a basis for testing speciation levels in *I.modestior* by involving neutral markers such as DNA sequences or chromosome morphology in order to obtain more insight into the evolution and diversification patterns of the studied populations.

Our study shows that bioacoustic analyses of Orthoptera are still useful to better understand geographic variation within species. Though the European fauna is fairly well studied compared to other continents, new species of Orthoptera from Europe have been continuously described in recent years (Kleukers et al. 2010; Ingrisch and Pavićević 2010; Iorgu and Iorgu 2010; Orci et al. 2010; Iorgu 2012; Szövényi et al. 2012; Kaya et al. 2012; Chobanov et al. 2014; Chobanov et al. 2015; Tilmans et al. 2016; Olmo-Vidal 2017). The latter particularly concerns the species-rich genera *Isophya* and *Poecilimon* that are still expected to hold further surprises. Discovering new cryptic taxa is, therefore, still realistic in Europe, especially as a result of integrated taxonomic studies. Altogether, our study shows that detailed bioacoustic analyses are useful to understand the geographic structure within species. As bioacoustics is often used in species identification, these data can also help to recognize the species in the field.

## Supplementary materials

Supplementary material 1 Table S1 Data type Table (excel file). Explanation note Detailed data on analysed specimens and song recordings. https://binary.pensoft.net/file/748775This dataset is made available under the Open Database License (http://opendatacommons.org/licenses/odbl/1.0/). The Open Database License (ODbL) is a license agreement intended to allow users to freely share, modify, and use this Dataset while maintaining this same freedom for others, provided that the original source and author(s) are credited.Slobodan Ivković, Dragan Chobanov, Laslo Horvat, Ionuț Ștefan Iorgu, Axel Hochkirch
